# Retrospective clinical audit of extraction cases treated with clear aligner therapy

**DOI:** 10.1186/s12903-025-07321-3

**Published:** 2025-12-04

**Authors:** Greeshma Jayapalan, Ismaeel Hansa, Raghdah Alzuhairy, Sandra Tai, Anand Marya, Samar M. Adel, Nikhillesh Vaiid

**Affiliations:** 1 Private practice, Dubai, United Arab Emirates; 2 Private practice, Durban, South Africa; 3 Graduate Orthodontics, Faculty of Dentistry, University of British Columbia, Vancouver, BC Canada; 4 Private practice, British Columbia, Canada; 5 Department of Orthodontics, Faculty of Dentistry, University of Puthisastra, No. 55, St 180, Phnom Penh, Phnom Penh, 12211 Cambodia; 6 Faculty of Dentistry, Alexandria University, Alexandria, Egypt; 7 Department of Orthodontics, European University College, Dubai, UAE; 8 Department of Orthodontics, Saveetha Dental College, Saveetha Institute of Medical and Technical Sciences, Chennai, Tamil Nadu India; 9 Department of Pediatric Dentistry and Orthodontics, Faculty of Dentistry, The University of Hong Kong, Hong Kong, China; 10 Department of Orthodontics, University of Illinois, Chicago College of Dentistry, Chicago, IL 60607 USA

## Abstract

**Objective:**

To evaluate the accuracy of clear aligners in premolar extraction cases by measuring the differences between predicted and achieved tooth movements.

**Materials and methods:**

The sample consisted of 32 patients undergoing extraction treatment with clear aligners, with a mean age of 21.2 (+-7.6) years. Discrepancies between achieved and predicted tooth movements were determined using paired t-tests and independent t-tests. The discrepancies per tooth group were assessed per dental arch and were evaluated for clinical significance (> 2 degrees; >0.5 mm).

**Results:**

Linear discrepancies that demonstrated clinical significance (> 0.5 mm) in the maxillary arch were the buccal-lingual and occlusal-gingival discrepancies for the central incisors (0.61 mm and 0.87 mm) and the buccal-lingual discrepancy of the first premolars (0.59 mm). The first and second molars in the mandibular arch showed buccal-lingual discrepancies of 0.51 mm and 0.65 mm, respectively. In comparison, the central (0.66 mm) and lateral incisors (0.57 mm) and first and second premolars (both 0.55 mm) showed clinically significant discrepancies in the occlusal-gingival direction. All angular discrepancies in the maxillary and mandibular dentition were statistically and clinically significant (> 2 degrees).

**Conclusions:**

Loss of torque and occlusal-gingival discrepancies of the upper and lower incisors were clinically significant. Unplanned tipping of teeth adjacent to the extraction site was also clinically significant. These factors should be considered and mitigated at the ClinCheck stage to aid efficiency.

## Introduction

Clear Aligners (CA) have become a mainstay of contemporary orthodontics. Over the past two and a half decades since its inception in 1998, Invisalign (Align Technology, Inc., Arizona, USA) has evolved to become a comprehensive orthodontic treatment modality with the benefit of greater esthetics and comfort [1]. However, to stake such a claim, acceptable and consistent treatment of difficult malocclusions is required. Extraction cases are generally more difficult to treat using clear aligners and require greater biomechanical considerations than non-extraction cases [2]. While numerous studies have examined the accuracy of clear aligners in non-extraction cases [3–7], studies documenting or investigating extraction cases have been few and far between, with the majority being case reports [8, 9]. A study by Chen et al., [10] comparing aligner and fixed appliances in premolar extraction cases using cephalometrics, found inferior outcomes and increased treatment durations using aligners. Dai et al. [11, 12] also attempted to study the accuracy of Invisalign in extraction cases. They found that first molar anchorage control and central incisor retraction were not achieved as predicted, with excessive mesial tipping of the molars, and distal tipping of the canines and incisors occurring. They also identified that the variables affecting accuracy were age, attachments used, and the degree of pretreatment crowding.

While the body of research on clear aligner treatment with extractions has been expanding, continued investigation remains essential to refine understanding of its limitations and to enhance the predictability of treatment simulations, thereby improving clinical efficiency. This research aims to test the prediction accuracy of Invisalign in extraction cases and identify significant discrepancies for specific teeth and movements. This insight empowers clinicians to make informed adjustments to their digital setups (e.g., ClinCheck), enabling them to overengineer specific tooth movements where necessary, thereby enhancing the predictability and efficiency of clear aligner treatments in extraction cases. Accordingly, the null hypothesis tested was that there would be no clinically significant differences in the three linear (mesial-distal, buccal-lingual, occlusal-gingival) and angular (tip, torque, rotation) discrepancies between predicted and achieved post-treatment tooth positions.

## Materials and methods

### Sample size estimation

The sample size was estimated using Gpower 3.1.9.4 based on the following parameters: statistical analysis = paired t-test, number of tails = 2, level of significance = 0.05, power of study = 80%, and effect size = 0.5 (medium effect). Therefore, the calculated sample size was 34.

### Sample

This retrospective single-center study was conducted in accordance with the declaration of Helsinki and approved by the institutional review board (IRB) of the European University College, Dubai (IRB No: EUC-IRB-18.11.15). The sample was obtained from a single orthodontist specialist practice, an Invisalign^®^ Diamond Elite provider located in Vancouver, Canada. The study only involved a non-identifying retrospective audit of patient records treated with a combination of clear aligners and premolar extractions hence informed consent was waived by the institutional review board of the European University College. The records of 59 consecutively treated Invisalign^®^ patients treated orthodontically with premolar extractions were initially reviewed. The records audited were from patients treated between January 2018 and December 2021.

The inclusion criteria were:

1. permanent dentition,
2. treated with G6 aligner protocol with weekly aligner changes
3. Class I and Class II malocclusion cases,
4. treated with 2 or 4 premolar extractions.

Patients were excluded based on:

1. incomplete records,
2. orthognathic surgery,
3. Impacted teeth (excluding third molars), and,
4. auxiliary appliances. (e.g., sectional fixed appliances, TADs, expanders, etc.)

The final sample that fitted the criteria consisted of 32 patients, 9 males and 23 females with a mean age of 21.2 years old (SD: 7.6; range: 11 to 40).

### Procedure

To obtain digital models of the predicted outcome, Stereolithographic (STL) models of the final stage of each patient’s virtual treatment plan were exported via the ClinCheck software. An intra-oral scan was taken after the initial set of aligners was completed to obtain digital models of the achieved outcome.

**Fig. 1:**
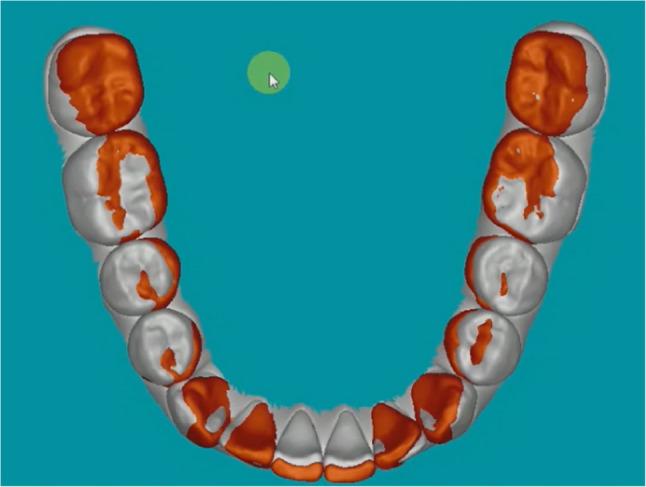
Digital models depicting the difference between predicted and achieved tooth positions in linear and angular dimensions

Both the intraoral scan (achieved model) and the virtual set-up (predicted model) were imported into the eModel Compare 9.0 software (GeoDigm Corporation, Falcon Heights, Minn). This software compares individual tooth positions between the two digital models and computes the difference between predicted and achieved tooth positions in linear and angular dimensions [13]. (Fig. 1)

The predicted model was segmented to isolate each tooth as a separate object and compared with the unsegmented achieved post-treatment models. Corresponding dental arches were first aligned globally, and then individual teeth from a segmented model were superimposed on analogous teeth of an unsegmented model using a best-fit algorithm to compute differences between tooth positions. The differences between the achieved and predicted position for each tooth were quantified for mesial‐distal, buccal‐lingual, occlusal‐gingival, tip, torque and rotational discrepancies. Linear differences were measured in millimeters, while angular differences were measured in degrees.

Baseline dental crowding was assessed using Little’s Irregularity Index on pre-treatment intraoral scans, yielding a mean irregularity of 5.4 ± 1.8 mm in the maxillary arch and 6.2 ± 2.1 mm in the mandibular arch across the studied cases.

Aspects of intra-examiner reliability were ordered into determining the reliability of an intra-examiner by measuring 20 randomly chosen intraoral scans on two occasions, two weeks apart. Inter-examiner reliability was evaluated separately by a second examiner measuring the same 20 scans. ICC was calculated for all measured parameters dealing with linear (mesial-distal, buccal-lingual, and occlusal-gingival displacements) and angular (tip, torque, and rotation) variables. The range for intra-examiner reliability ICC values was found to be 0.91 to 0.97, while the inter-examiner reliability ICC range was found to be 0.88 to 0.95; both values showing excellent agreement according to established criteria.

In order to compare expected and actual positions of teeth, we performed surface-based superimposition of post- and pretreatment intraoral scans using eModel Compare 9.0 (GeoDigm Corporation, Falcon Heights, Minn). The iterative closest point algorithm was used with stable posterior teeth—i.e., lingual cusps of first premolars and molars, and second molar distal surfaces—as reference areas in order to limit registration error, particularly in the mandibular arch where few fixed anatomical landmarks are present. Unlike other studies utilizing CBCT for better registration accuracy (e.g., Dai et al.), our process avoids unnecessary exposure to radiation while not compromising clinically acceptable precision for crown-based measurements. Our process has already been tested and validated in previous aligner outcome studies and is applicable for discrepancies in linear and angular crown movement measurements [13].

Analogous teeth were averaged, and descriptive statistics were computed per tooth type for each dental arch. The descriptive statistics represented the predicted-achieved differences for central incisors, lateral incisors, canines, first premolars, second premolars, first molars and second molars for the maxilla (Table 1) and the mandible (Table 2).

**Table 1:** Maxillary arch: discrepancies for linear and angular movements per tooth group

Tooth group (*n*)	Mesial- Distal (mm)		Buccal- Lingual (mm)		Occlusal- Gingival (mm)		Tip (°)		Torque (°)		Rotation (°)		
	Mean	SD	Mean	SD	Mean	SD	Mean	SD	Mean	SD	Mean	SD	
Central incisor (32)	0.15	± 0.09	0.61*	± 0.35	0.87*	± 0.43	2.41*	± 1.37	5.55*	± 3.59	2.05*	± 1.24	
Lateral incisor (32)	0.32	± 0.17	0.38	± 0.25	0.45	± 0.25	3.84*	± 2.57	4.38*	± 3.34	3.10*	± 2.06	
Canine (32)	0.36	± 0.20	0.26	± 0.16	0.30	± 0.14	5.55*	± 2.30	2.51*	± 1.48	3.59*	± 2.88	
First premolar (8)	0.31	± 0.14	0.59*	± 0.22	0.26	± 0.17	3.46*	± 0.96	3.11*	± 2.00	3.79*	± 1.46	
Second premolar (24)	0.28	± 0.20	0.39	± 0.32	0.47	± 0.25	3.73*	± 1.83	3.11*	± 2.00	3.43*	± 2.51	
First molar (32)	0.36	± 0.32	0.45	± 0.33	0.35	± 0.22	4.32*	± 2.26	2.48*	± 1.60	2.61*	± 2.07	
Second molar (28)	0.49	± 0.28	0.49	± 0.34	0.41	± 0.24	5.22*	± 2.92	3.71*	± 2.64	3.00*	± 2.06	

*Denotes clinically significant discrepancies

**Table 2:** Mandibular arch: discrepancies for linear and angular movements per tooth group

Tooth group (*n*)	Mesial- Distal (mm)		Buccal- Lingual (mm)		Occlusal- Gingival (mm)		Tip (°)		Torque (°)		Rotation (°)	
	Mean	SD	Mean	SD	Mean	SD	Mean	SD	Mean	SD	Mean	SD
Central incisor (32)	0.18	± 0.07	0.37	± 0.34	0.66*	± 0.49	2.60*	± 1.78	6.52*	± 3.57	2.87*	± 1.84
Lateral incisor (30)	0.19	± 0.10	0.27	± 0.21	0.57*	± 0.38	3.51*	± 1.99	5.39*	± 2.94	2.37*	± 1.53
Canine (32)	0.27	± 0.19	0.27	± 0.12	0.28	± 0.18	2.92*	± 2.20	2.70*	± 2.34	3.31*	± 2.09
First premolar (18)	0.33	± 0.24	0.40	± 0.27	0.55*	± 0.29	4.10*	± 2.36	6.02*	± 3.82	4.24*	± 3.23
Second premolar (28)	0.33	± 0.24	0.35	± 0.24	0.55*	± 0.34	4.68*	± 2.90	6.02*	± 3.82	4.06*	± 2.35
First molar (32)	0.32	± 0.22	0.51*	± 0.35	0.41	± 0.20	2.15*	± 1.34	4.40*	± 2.19	3.06*	± 2.52
Second molar (30)	0.31	± 0.20	0.65*	± 0.47	0.40	± 0.28	4.21*	± 3.05	5.37*	± 3.15	2.31*	± 1.89

*Denotes clinically significant discrepancies

### Statistical analysis

Data exploration and cleaning were initially performed to investigate missing values and incorrect data entry. The normality distribution of the data was then assessed using Kolmogorov-Smirnov and Shapiro-Wilk tests, and the data was found to be normally distributed (*P* > 0.05). Hence, the statistical analyses performed were descriptive statistics, paired t-test, and independent t-test. The descriptive statistics present the means and standard deviations (SD). Paired t-test was used to determine intra-arch mean differences, and independent t‐test was used to determine inter-arch mean differences between predicted and achieved tooth positions. The *P*-value of less than 0.05 was the threshold used to determine statistically significant differences. Clinical significance for accuracy of achieving predicted tooth positions was set for linear movements at greater than 0.5 mm and for angular movements at greater than 2.0° according to discrepancies determined by Grunheid et al. [14], which is based on the American Board of Orthodontics scoring system. All statistical analyses were performed using the Statistical Product and Service Solution (SPSS) version 29.

## Results

Table 1 presents the discrepancies for linear and angular movements per tooth group in the upper arch. Linear discrepancies that demonstrated clinical significance (> 0.5 mm) in the maxillary arch were the buccal-lingual and occlusal-gingival discrepancies for the central incisors (0.61 mm and 0.87 mm, respectively) and the buccal-lingual discrepancy of the first premolars (0.59 mm). All angular discrepancies demonstrated clinical significance (> 2.0°) in both the maxillary and mandibular dental arches. The tooth with the greatest angular discrepancy in the maxillary arch for Tip was the upper canines (5.55°), followed closely by the second molar (5.22°). The upper central incisor (5.55°) and lateral incisor (4.38°) showed the greatest discrepancies for torque. Rotational discrepancies in the upper arch were much more consistent between the different teeth, with the upper first premolar being the greatest (3.79°).

Table 2 presents the discrepancies for linear and angular movements per tooth group in the lower arch. In the mandibular arch, the first and second molars showed buccal-lingual discrepancies of 0.51 mm and 0.65 mm, respectively. In comparison, the central (0.66 mm) and lateral incisors (0.57 mm) and the first and second premolars (both 0.55 mm) showed clinically significant discrepancies in the occlusal-gingival direction. In the mandible, the greatest discrepancy for Tip was the second premolar (4.68°), followed by the second molar (4.21°) and first premolar (4.10°). The central incisors showed the greatest discrepancy for torque (6.52°). The rotational discrepancies were also more consistent between the different teeth; however, the first and second premolars were the least accurate (4.24° and 4.06°, respectively).

To further quantify vertical side effects and support the observation of the “roller coaster effect,” the Buccal Axial Occlusal Plane (BAOP) angle was measured between the bilateral first premolars in pre- and post-treatment intraoral scans. The mean BAOP angle increased significantly from 2.3° ± 1.2° at baseline to 6.4° ± 2.1° post-treatment (*p* < 0.05). This angular change reflects a vertical bowing of the occlusal plane, suggesting relative extrusion of premolars and/or loss of posterior anchorage, consistent with previously described “roller coaster effects” in aligner therapy (Qian et al., 2024)). Observed effect sizes (Cohen’s *d*) for the primary outcome measures ranged between 0.42 and 0.68, indicating small-to-moderate effects. Post-hoc power analyses based on these effect sizes confirmed an achieved power, reflecting adequate but slightly reduced statistical strength relative to the initial calculation.

## Discussion

Linear discrepancies that demonstrated clinical significance (>0.5 mm) in the maxillary arch were only the Buccal-Lingual and Occluso-Gingival discrepancy for the central incisors and the Buccal-Lingual discrepancy of the first premolars. This implies generally good linear prediction in the upper arch. In the mandibular arch, the first and second molars showed significant Buccal-lingual discrepancies, while the central and lateral incisors, and first and second premolars showed clinically significant discrepancies in the Occlusal-Gingival direction. Overall, when looking at crown movements, it seemed the linear predictions were generally accurate with the exception of Occlusal-Gingival movements. However, when we attempted to evaluate the accuracy of angular and root movements, we found that none of the angular discrepancies met clinical expectations, as all 42 angular discrepancies were clinically significant (>2°), indicating that while the crowns of the teeth moved relatively well, the roots did not follow suit, resulting in large discrepancies in the Tip and Torque angular movements Rotational discrepancies were primarily found in rounded teeth such as the premolars, which previous studies have corroborated [3, 5, 15, 16]. 

The aligners failed to prevent the “rollercoaster effect,” which is caused by tipping movements instead of the desired bodily movement, despite the use of the G6 protocol. This phenomenon is usually seen in fixed appliances during retraction on lighter wires but now also reflects the inherent lack of rigidity of aligners and failure to obtain bodily movement during retraction. It is essential to over-engineer incisor torque and intrusion in both upper and lower arches to prevent excessive retroclination of the incisors and deepening of the bite. The teeth adjacent to the extraction sites (canines, premolars and molars) showed unintended tipping in excess of 4^o^. Therefore, root inclinations adjacent to the extraction sites require over-engineering by additional Tip by around 5–6^o^. Feng et al. [17] however, suggested greater overcorrection by recommending additional distal crown tip for the second premolar (10.73°), first molar (9.83°) and second molar (7.18°), and additional 8.59° of mesial tip for the canine. Palone et al. [15] suggested that 20% overcorrection should be prescribed for movements such as tip, torque and rotations. Extruding the premolars and intruding the incisors and finishing the Clincheck with a 0 mm overbite may also be recommended as suggested by Kravitz et al. [18] This can be described traditionally as adding a reverse curve of Spee in the lower arch and accentuated curve of Spee in the upper arch.

When comparing our results to similar non-extraction studies, control of angular movements is one of the most challenging movements [19]. Where in non-extraction treatment rotations seem to be most problematic [3], in extraction treatment, the tip and torque discrepancies were much larger than that of rotational ones. The study by Dai et al., [11] where they compared achieved and predicted tooth movements of maxillary first molars and central incisors in premolar extraction cases treated with Invisalign, is the only previous study similar to ours. We found similar results with regard to molar tipping, with both studies reporting a 5^o^ discrepancy. Both studies also found approximately a 5^o^ discrepancy of Torque for the upper incisors.

A study by Tang et al. (2023) on anchorage management in clear aligner therapy is elucidated here, the result of an investigation into relative anchorage loss (RAL) in mandibular premolar extraction cases which provides a clinical analogy. Tang et al. reported an average RAL of first premolar extraction at 25% and a second premolar extraction at 40%, thus indicating the different ability of anchorage preservation depending on extraction location. Although focusing on maxillary tooth movements, the angular and linear discrepancies observed—particularly in torque and tip deviations in anterior segments—reveal similar anchorage considerations. Comparison of these data only amplifies the need for individualized anchorage strategies, suggesting that RAL-based planning frameworks, as proposed by Tang et al., will improve predictability and control in aligner-based treatment protocols.

Our findings accord with the concerns mentioned by Qian et al. (2024), who observed that transparent aligners may contribute to an excessive buccal occlusal plane curvature termed the “roller coaster effect” particularly in premolar extraction instances. The observed rise in the BAOP angle in our group further confirms this tendency, showing vertical bowing of the posterior occlusal plane. This condition presumably results from insufficient posterior anchoring control and extrusion of premolars during space closure. The findings highlight the necessity of employing vertical anchorage solutions, such optimal attachments or supplementary mechanics, to reduce occlusal instability and maintain arch form during aligner treatment.

Thoughtful planning and treatment strategies should be implemented to mitigate the limitations of clear aligners in premolar extraction cases. Tipping of teeth adjacent to the extraction site, loss of torque of the anterior teeth, and excessive deepening of the bite may occur if not accounted for or if auxiliary appliances are not used [20]. Despite what the simulations may indicate, mechanics should be tailored toward over-engineering of the desired outcome.

A major limitation of planning tooth movements using Invisalign is that ClinCheck is simply a visual representation of the force systems rather than a predictor of final tooth position [21–23].

The following suggestions for over-engineering the ClinCheck G6 protocol can be used to obtain better outcomes:

- Finish with a 0 mm overbite by intruding upper and lower incisors and extruding premolars.
- Place an additional 6–7 degrees of additional lingual root torque to the upper and lower incisors.
- Place an additional 5–6 degrees of further overcorrection to converge the roots of teeth adjacent to the extraction spaces [24, 25].

## Limitations

The initial sample size estimation assumed a moderate effect size due to the lack of comparable prior studies. However, the observed effect sizes were calculated post hoc and indicated small-to-moderate values, which should be considered when interpreting the generalizability of our findings. The final sample size (*n* = 32) was slightly smaller than the priori calculated requirement (*n* = 34), which may limit statistical power and should be considered when interpreting the results.

## Conclusion

- Loss of torque and occlusal-gingival discrepancies of the upper and lower incisors were both observed. Likewise, unplanned tipping of teeth adjacent to the extraction site were clinically significant.
- Torque control was not achieved as predicted in extraction cases and tipping was more observed.

## Data Availability

The data supporting this study’s findings are available from the corresponding author upon reasonable request.
